# Quality and Variability of Patient Directions in Electronic Prescriptions in the Ambulatory Care Setting

**DOI:** 10.18553/jmcp.2018.17404

**Published:** 2018-07

**Authors:** Yuze Yang, Stacy Ward-Charlerie, Ajit A. Dhavle, Michael T. Rupp, James Green

**Affiliations:** 1 Surescripts, Arlington, Virginia.; 2 Midwestern University, Glendale, Arizona.

## Abstract

**BACKGROUND:**

The prescriber’s directions to the patient (Sig) are one of the most quality-sensitive components of a prescription order. Owing to their free-text format, the Sig data that are transmitted in electronic prescriptions (e-prescriptions) have the potential to produce interpretation challenges at receiving pharmacies that may threaten patient safety and also negatively affect medication labeling and patient counseling. Ensuring that all data transmitted in the e-prescription are complete and unambiguous is essential for minimizing disruptions in workflow at prescribers’ offices and receiving pharmacies and optimizing the safety and effectiveness of patient care.

**OBJECTIVES:**

To (a) assess the quality and variability of free-text Sig strings in ambulatory e-prescriptions and (b) propose best-practice recommendations to improve the use of this quality-sensitive field.

**METHODS:**

A retrospective qualitative analysis was performed on a nationally representative sample of 25,000 e-prescriptions issued by 22,152 community-based prescribers across the United States using 501 electronic health records (EHRs) or e-prescribing software applications. The content of Sig text strings in e-prescriptions was classified according to a Sig classification scheme developed with guidance from an expert advisory panel. The Sig text strings were also analyzed for quality-related events (QREs). For purposes of this analysis, QREs were defined as Sig text content that could impair accurate and unambiguous interpretation by staff at receiving pharmacies.

**RESULTS:**

A total of 3,797 unique Sig concepts were identified in the 25,000 Sig text strings analyzed; more than 50% of all Sigs could be categorized into 25 unique Sig concepts. Even Sig strings that expressed apparently simple and straightforward concepts displayed substantial variability; for example, the sample contained 832 permutations of words and phrases used to convey the Sig concept of “Take 1 tablet by mouth once daily.” Approximately 10% of Sigs contained QREs that could pose patient safety risks or workflow disruptions that could necessitate pharmacist callbacks to prescribers for clarification or other manual interventions.

**CONCLUSIONS:**

The quality of free-text patient directions in e-prescriptions can vary dramatically. However, more than half of all patient directions sent in the ambulatory setting can be categorized into only 25 Sig concepts. This suggests an immediate, practical opportunity to improve patient safety and workflow efficiency for both prescribers and pharmacies. Recommendations include implementing enhancements to Sig creation tools in e-prescribing and EHR software applications, adoption of the Structured and Codified Sig format supported by the current national e-prescribing standard, and improved usability testing and end-user training for generating complete and unambiguous patient directions. Such quality improvements are essential for optimizing the safety and effectiveness of patient care as well as for minimizing workflow disruptions to both prescribers and pharmacies.

**What is already known about this subject**

- Electronic prescribing (e-prescribing) provides numerous benefits in patient care and workflow efficiency, including the mitigation of handwriting illegibility problems as the industry transitions from traditional paper prescriptions; however, quality-improvement opportunities remain that need to be addressed in order for providers and patients to experience the full benefits of this technology.
- Patient directions (Sig) can be written with myriad text string variations while conveying the same core concepts, due in part to the several hundred e-prescribing software applications, each with its own unique Sig-writing process.
- In addition to the numerous possible textual variations that may be used to communicate 1 distinct Sig concept, some variations may also introduce quality challenges that can cause confusion, misinterpretations, and workflow disruptions when an e-prescription is received by the pharmacy.

**What this study adds**

- The variability encountered in e-prescription Sig text strings in the ambulatory care setting were quantified and characterized.
- Quality issues encountered in Sig text strings that may cause workflow disruptions or introduce patient safety risks were identified and classified.
- Recommendations were derived for health care industry stakeholders for prescribers and pharmacies related to standardization and quality improvements for enhancing interoperability, workflow efficiency, and overall patient care.

Owing to its prospects for producing increased efficiency, reduced costs, and improved quality of care, electronic prescribing (e-prescribing) has become one of the most widely used components of the U.S. health care information technology infrastructure. Furthermore, e-prescribing has also been advanced by legislation and mandates such as the Health Information Technology and Clinical Health Act and Medicare Improvements for Patients and Providers Act, as well as billions of dollars in incentives for infrastructure developments to meet Meaningful Use requirements as directed by the Centers for Medicare & Medicaid Services.^1-4^

However, as with any technology, the benefits from e-prescribing are directly dependent on the manner in which it is implemented and used, and prior research has demonstrated that e-prescribing can elevate the risks of some errors or even introduce new quality challenges.^5-8^ Few components of a prescription order are more important for ensuring safe and effective pharmaceutical care than the prescriber’s directions for how patients administer their medications, as communication of clear and complete instructions is essential to ensure proper prescription labeling, appropriate pharmacist counseling, and optimal medication usage.^9,10^ These patient instructions, or Sigs (*signetur*), are communicated within e-prescriptions via a 140-character free-text field in the National Council for Prescription Drug Programs (NCPDP) SCRIPT Standard version 10.6.^11^ Although the Sig may also be communicated as an independent structured and codified data segment in the current standard, various challenges have limited its implementation as such; hence, most e-prescription Sigs continue to be transmitted in the free-text data field.^12^

Previous studies have revealed the potential for significant variability in prescriber-generated Sig free-text strings.^13-15^ For example, a seemingly straightforward Sig concept such as “Take one capsule once every day” can be expressed in numerous ways, some of which may be ambiguous or confusing and necessitate callbacks to prescribers for clarification to reduce the potential for patient safety risks.^10,16^ Thus, the objectives of this study were to quantify and characterize variations in free-text Sig strings, evaluate the prevalence of quality-related events (QREs) leading to interpretation problems or workflow disruptions at receiving pharmacies, and develop recommendations for improving current e-prescribing practices relative to this quality-sensitive field.

## Methods

We analyzed free-text Sigs in new e-prescription (NewRx) messages transmitted by prescribers in the ambulatory care setting through a national health information network to community pharmacies of a large national pharmacy chain.

A random sample of 25,000 e-prescriptions was drawn from 831,590 NewRxs transmitted on July 21, 2014, to all stores in the chain. The study sample size was determined to be representative of the 831,590 NewRx prescriptions volume on that day with a margin of error of 0.9% at a confidence level of 99.6% using the Raosoft sample size calculator.^17^ Extracted data were de-identified by the pharmacy chain before being provided to the investigation team in accordance with the Health Insurance Portability and Accountability Act Privacy Rule at 45 CFR §164.514. No personally identifiable patient-specific data were made available to the investigation team for analysis. Analysis of e-prescription Sig content was conducted in 2 phases between January 12, 2015, and August 29, 2016.

### Phase 1: Classification of Unique Sig Concepts

In Phase 1, the research team initially removed duplicates from the data sample to identify unique Sig text strings. Two residency-trained clinical pharmacists with more than 5 years of ambulatory care experience (YY and SWC) classified each unique text string using a Sig Content Concept Classification Scheme (SCCCS) that had been created for this study (Appendix, available in online article). A third licensed pharmacist (AAD) who was experienced in community pharmacy practice served as the final adjudicator when consensus could not be reached between the 2 primary reviewers.

The SCCCS was created by the research team (YY, SWC, AAD, and MTR) in collaboration with an expert advisory panel. The research team and advisory panel members represented 4 medical and pharmacy schools, a national retail pharmacy chain, an independent consultant, and a national health information network. All panelists had extensive previous experience with e-prescribing technologies and familiarity with the NCPDP SCRIPT Standard version 10.6.

### Phase 2: Classification of Sig Quality Issues

In Phase 2, Sig text strings were independently reviewed and assessed by 2 pharmacy technicians for potential QREs. QREs were defined as any incomplete or ambiguous Sig information that would likely cause interpretation problems leading to workflow disruptions at receiving pharmacies because they would require further clarification with prescribers before the prescription could be processed and dispensed. Examples of QREs included key informational elements (e.g., dose or frequency) that were missing, ambiguous, contradictory, or unintelligible. Both reviewers were certified pharmacy technicians and had more than 2 years of experience interpreting and processing prescriptions in community practice settings. Before the review, the reviewers were extensively trained over 2 months, followed by individual assessments for ensuring proficiency in classifying QREs using a QRE classification scheme developed by the research team. When consensus could not be reached between the 2 primary reviewers on QRE classification, a residency-trained pharmacist with ambulatory and acute care experience (YY) served as the final adjudicator.

## Results

The 25,000 e-prescriptions analyzed in the study were issued by 22,152 ambulatory care prescribers practicing in 48 states, the District of Columbia, and Puerto Rico. These e-prescriptions were generated from 501 e-prescribing software applications, including both standalone applications as well as those integrated within electronic health record (EHR) systems. The top 20 of these e-prescribing systems accounted for 62.7% of the NewRx volume.

### Sig String Variations and Unique Concepts (Phase 1)

A total of 3,797 unique Sig concepts were identified in the 14,463 unique Sig text strings that resulted after eliminating duplicates from the original sample of 25,000 NewRx Sigs. The 2 pharmacist reviewers demonstrated moderately strong agreement in their assignments of Sig strings to unique Sig concepts, as evidenced by a calculated kappa coefficient of κ = 0.76.^18^ Of the 3,797 unique Sig concepts in the SCCCS, the 25 most frequently observed concepts accounted for 12,798 (51.2%) Sig text strings (Table 1). In contrast, 3,223 concepts were observed in 3 or fewer e-prescription Sig strings and accounted for a total volume of only 4,091 (16.0%) e-prescriptions in the sample of NewRx Sigs.

**TABLE 1:** Most Common Sig Concepts in e-Prescription Sig Text Strings

Sig Concept	n	NewRx%	Text String Variations	Text String Variation Example 1^a^	Text String Variation Example 2	Text String Variation Example 3
Take 1 tablet by mouth once daily	5,202	20.8	832	*take 1 tablet by oral route every day*	*1 (one) Tablet, Oral, QD*	*Take one (1) tablet by mouth once a day*
Take 1 tablet by mouth twice daily	1,542	6.2	435	*Take 1 tablet (500 mg total) by mouth 2 (two) times daily*	*Take 1 tab(s) bid orally*	*twice a day, 1 tab po bid*
Take 1 tablet by mouth once daily for X duration	927	3.7	298	*Take one tablet QD × 30 days.*	*take by oral route every day × 10 days*	*Take 1 tablet once a day Orally 90 days*
Take 1 tablet by mouth once daily at bedtime	754	3.0	307	*Take one tab QHS*	*Take one pill by mouth daily at bedtime*	*Take 1 tablet(s) by mouth 1 time a day at bedtime*
Take 1 capsule by mouth once daily	667	2.7	198	*take 1 capsule (12.5 mg) by oral route once daily*	*Take 1 cap po qd*	*Take one capsule every day*
Take 1 tablet by mouth twice daily for X duration	479	1.9	279	*Take 1 tablet(s) twice a day by oral route for 90 days.*	*Take one tablet twice daily for 3 days*	*1 tablet 2x/day for 7 days*
Take 1 tablet by mouth 3 times daily	272	1.1	138	*Oral take 1 tablet 3 times a day*	*take 1 tablet (800MG) by oral route 3 times every day*	*Take 1 tab(s) TID orally*
Use/take as directed/instructed	266	1.1	66	*Use per instructions*	*Use as directed.*	*Take as directed*
Apply product topically to the affected area twice daily	237	0.9	141	*1 App 2 times per day*	*1 (one) Cream, External, BID*	*0.1 % TOP BID*
Take 1 tablet by mouth once daily in the morning	224	0.9	122	*1 daily in AM*	*1 TAB ORAL Once daily in am*	*1 tab(s) PO qam*
Take 1 capsule by mouth twice daily	220	0.9	96	*Take one capsule orally twice daily*	*Give 1 Cap by mouth 2 times daily.*	*Take one (1) delayed release capsule Twice a Day*
Apply topically to the affected area twice daily	197	0.8	114	*twice a day, Apply twice daily to affected area*	*Apply twice a day to affected areas on feet.*	*Apply TAA BID*
Take 1 tablet by mouth twice daily with food	193	0.8	121	*Take 1 tablet orally Twice a day with food*	*Take one tablet by mouth twice daily with food.*	*Take 1 tablet(s) by mouth bid with meals*
Use 2 sprays in each nostril once daily	179	0.7	107	*Use 2 sprays in each nostril once daily*	*Two spray per nostril daily.*	*Take 2 spray in both nostrils given once a day.*
Take 2 tablets by mouth once daily	159	0.6	105	*2 tablet PO qDay*	*Take 2 tablets orally every day*	*Two tabs PO QD*
Take 2 tablets by mouth once on the first day, then take 1 tablet by mouth once daily for 4 days	157	0.6	79	*Take by mouth 2 tablets on day 1, then 1 tablet daily for 4 days*	*#2 po day #1, then #1 po days 2-5*	*two tablets on day one, then one tablet daily × 4 days*
Take 1 tablet by mouth once daily as directed	156	0.6	35	*Take 1 tablet every day as directed*	*take 1 tablet Oral once a day as directed i po q d*	*Take as directed 1 PO Daily Orally*
Take 1 capsule by mouth once daily for X duration	148	0.6	69	*TAKE 1 CAPSULE DAILY 30 days*	*Take One capsule by mouth every day for 8 Weeks*	*1 cap PO Daily,x30 day*
Take 1 tablet by mouth every 12 hours	146	0.6	43	*Take 1 tablet every 12 hrs Orally*	*take 1 tablet (500MG) by oral route every 12 hours*	*1 tab(s) orally every 12 hours*
Take 1 tablet by mouth once daily in the evening	136	0.5	63	*Take 1 tablet(s) by mouth each evening*	*Take 1 tablet (40 mg total) by mouth every evening.*	*1 Tablet(s) PO QPM*
Take 1 tablet by mouth 3 times daily for X duration	116	0.5	86	*One pill three times daily for 10 days*	*take 1 tablet po tid for 7 days*	*Take 1 tab TID × 14 days*
Take 1 tablet by mouth once daily at bedtime for X duration	107	0.4	68	*Take 1 pill by mouth QHS (nightly) X 3 Months (90d)*	*1 Tablet At Bedtime for 1 month*	*1 tablet qhs Orally 90 Days*
Take 1 tablet by mouth 3 times daily as needed	106	0.4	64	*Take 1 tablet as needed tid Orally*	*Take 1 tablet by mouth three times a day as needed*	*1 tablet PO TID PRN*
Take 1 capsule by mouth twice daily for X duration	105	0.4	78	*Take 1 Capsule oral bid (twice a day) for 10 days*	*1 capsule BID Orally 30 day(s)*	*Take 1 capsule by mouth twice daily for 5 days. FOR 5 DAYS.*
Take 1 capsule by mouth 3 times a day	103	0.4	54	*1 capsule tid*	*take 1 capsule by oral route 3 times every day*	*Take one (1) capsule Three Times a Day*
**TOTAL**	**12,798**	**51.2**				

*^a^All example Sig text strings are presented exactly as they appeared in the original e-prescription.*

*AM = morning; BID = twice daily; d = days; po = by mouth; PRN = as needed; qam = every day before noon; qd = every day; qDay = every day; qhs = every night at bedtime; qpm = every afternoon; Sig = prescriber’s directions to the patient; TAA = to affected areas; TID = three times a day; TOP = topical.*

Closer examination of these infrequently used Sig concepts revealed many relatively complex patient instructions that differed markedly from the much simpler concepts that were observed in the majority of NewRx messages. For example, the Sig concept of “Take 1 tablet by mouth 3 times a day for two days, then take 1 tablet by mouth twice a day for the next two days, and then take 1 tablet by mouth once daily for 2 days” occurred in only 2 e-prescriptions in the sample; 1 in which the concept was represented in the Sig string as “*1 po tid x3 days, 1 po bid X2 days, 1 po qd X2 days*” and the other that was represented as “*take 1 tablet by oral route 3 times every day on day1,2; then 1 tab BID on day3,4; then 1 tab daily on day5,6*.” Another example of a complicated Sig concept was “Instill 1 drop into the right eye every 15 minutes for the first hour and then every hour for the remainder of the first day, then instill 1 drop four times daily,” which occurred in only a single e-prescription containing the Sig string “*1 gtt Q15 min OD for the first hour then Q1hr for the rest of that day, then QID*.”

### Quality-Related Events in Sigs (Phase 2)

Of the 25,000 NewRx e-prescription Sigs that were analyzed, 2,516 (10.1%) contained at least 1 QRE that reviewers agreed would likely produce confusion or workflow disruptions at receiving pharmacies that may require contact with the prescriber to clarify or correct (κ = 0.72). The distribution of QREs identified in the Sig text strings is summarized in Table 2.

**TABLE 2:** Quality-Related Events in e-Prescription Free-Text Sig Strings

Sig QRE	Examples^a^	n (%)
Missing dose	*Drug Description: Atenolol 50 MG tablet* *Sig: “BID*	1,622 (53.6)
Missing site for relevant medications	*Drug Description: Acanya 1.2 %-2.5 % topical gel* *Sig: “TOP QAM”*	514 (17.0)
Contains non-Sig information meant for other designated fields	*Drug Description: FOSAMAX 70 MG TABS* *Sig: “Please enter as generic Alendronate”*	479 (15.8)
Sig conflicts with prescribed drug description	*Drug Description: Metronidazole 250 MG Oral Tablet* *Sig: “5 mL, oral, three times daily”*	168 (5.6)
Conflicting, ambiguous, or nonsensical Sig	*Drug Description: Aldactone 25 MG TABS* *Sig: “daily, M W Friday (not daily)”*	94 (3.1)
Difficult-to-read directions with unnecessary repetitions	*Drug Description: Clomid 50 mg oral tablet* *Sig: “2 tablet ORAL Daily,x5 day(s),Instr:2 tablets-100 mg-daily on days 5-9 of cycle”*	67 (2.2)
Missing frequency	*Drug Description: quinapril 10 mg tablet* *Sig: “Take 1 tab 1 orally”*	61 (2.0)
Non-English/uninterpretable	*Drug Description: Metfromin HCL 1000 MG TABS* *Sig: “tome una tableta 2 veces al dia”*	17 (0.6)
Incomplete or truncated Sig	*Drug Description: Metformin ER 500 MG PO TB24* *Sig: “Take 2 tablets in the am and take 2 tablets in”*	3 (0.1)
**TOTAL**		**3,025 (100.0)^b^**

*^a^All example Drug Description and Sig text strings are presented exactly as they appeared in the original e-prescription.*

*^b^Some Sigs contained more than 1 QRE.*

*BID = twice daily; PO = by mouth; QAM = every day before noon; QRE = quality-related event; Sig = prescriber’s directions to the patient.*

Additional QRE analysis was conducted on Sig concepts present in at least 20 NewRx messages. Although the overall highest volume of QREs were associated with Sig concepts for the most commonly prescribed products, e.g., usually oral tablets and capsules, the 3 Sig concepts with the highest rates of QRE were related to instructions for administering topical medication formulations such as creams or ointments, as illustrated in Table 3. The majority of these Sigs were missing an instructed dose amount, followed by the omission of a specified site of administration.

**TABLE 3:** Sig Concepts with the Highest QRE Rates

Sig Concept	QRE Classification	Examples	n (%)
Apply product to the affected area twice daily	Missing dose, missing site, and/or contains non-Sig information	*Drug: triamcinolone (KENALOG) 0.1 % cream* *Sig: “Apply topically 2 times daily. PUT ON FILE ONLY”*	235 (92.9)
	No QRE	*Drug: triamcinolone acetonide 0.1 % topical ointment* *Sig: “apply by TOPICAL route 2 times every day a thin film to the affected skin areas”*	18 (7.1)
	**TOTAL**		**253 (100.0)**
Apply product to affected area three times daily for X duration	Missing dose and/or missing site	*Drug: BACTROBAN 2% Ointment* *Sig: “Apply Ointment 3 times a day External FOR 10 days”*	34 (91.9)
	No QRE	*Drug: erythromycin 5 mg/gram (0.5 %) ointment* *Sig: “Apply thin ribbon to right eye tid for 5-7 days”*	3 (8.1)
	**TOTAL**		**37 (100.0)**
Apply product to the affected area twice daily as needed	Missing dose and/or missing site	*Drug: Hydrocortisone 1 % External Cream* *Sig: “1 (one) Cream, External, two times daily, as needed”*	20 (90.9)
	No QRE	*Drug: triamcinolone acetonide 0.1 % topical cream* *Sig: “Apply thin film to the affected areas by topical route BID PRN”*	2 (9.1)
	**TOTAL**		**22 (100.0)**

*^a^ All example Drug Description and Sig text strings are presented exactly as they appeared in the original e-prescription.*

*BID = twice daily; PRN = as needed; QRE = quality-related event; Sig = prescriber’s directions to the patient; tid = three times a day.*

## Discussion

The safety and effectiveness of prescription pharmaceutical care relies on pharmacists unambiguously interpreting and implementing the therapeutic intent of prescribers. While e-prescribing has mitigated certain barriers to clear communication, notably handwriting illegibility, opportunities remain for this technology to achieve its full potential for improving the quality of patient care. The Sig has long been recognized as a particularly quality-sensitive component of prescription orders, and the free-text Sig field in e-prescriptions maintains several limitations, as significant variability in how Sig concepts are expressed in free-text remains a concern for patient safety and workflow efficiency.

In our analysis of ambulatory NewRxs, 3,797 unique Sig concepts were found to be represented in 14,463 unique Sig text strings. This finding suggests significant opportunities for improving standardization, as more than half of the samples were classified under only 25 (0.7%) distinct concepts. Conversely, nearly 85% of Sig concepts were observed so infrequently (i.e., 3 or fewer times) that they collectively represented only 16% of the total sample. Additionally, unique Sig concepts were often represented by multiple free-text string variations. In 1 example, 832 separate variants were found to convey one of the least complex and most frequently used Sig concepts, “Take 1 tablet by mouth once daily.”

Such extreme variation in text strings, all of which were presumably intended to convey the same patient instructions, introduces substantial potential for syntax that may be sufficiently unclear for the pharmacist and patient, which could warrant manual interventions in order to reduce misinterpretation risks. The manual interventions required to clarify, reformat, or retranscribe the Sig text can subsequently produce interruptions, distractions, and inefficiencies for pharmacy personnel and represents time that could be better spent on direct patient care and counseling.

In addition to showing wide variance in how Sig concepts are communicated in Sig strings, more than 10% of Sigs contained quality problems that could pose direct threats to the quality and safety of patient care. For example, a Sig such as “*daily, M W Friday* (*not daily*)” is not only incomplete due to a missing dose value, but also confusing due to its contradictory instructions. At best, such Sigs that contain missing, ambiguous, conflicting, or nonsensical information contained in these Sig strings would again create productivity and efficiency problems, especially for high-volume pharmacies and prescriber offices or clinics where calls for clarification interrupt their daily practice. At worst, such Sigs present a source for potential dispensing or counseling errors that result in adverse patient outcomes.

The multitude of e-prescribing applications that prescribers use can differ significantly in their Sig construction features, which contributes to the variations in Sig text strings observed in this analysis. For example, most EHR applications provide end-users with Sig-builder tools that allow users to select each element in the Sig, including the administration action (e.g., “take” or “instill”), dose amount, route of administration, site of administration (when applicable, such as for topical products), administration frequency or timing, and various auxiliary instructions such as duration or indication. These Sig-builders may display a series of options for selecting each Sig component in a certain order, with a fully constructed text string that concatenates the discrete fields together and presents the completed Sig within the context of the entire e-prescription for provider verification before transmitting. However, certain applications may also allow prescribers to append additional free-text instructions to the Sigs constructed from the Sig-builder tool.^19^ In some cases, this subsequently introduces opportunities for further variability and information that may be irrelevant, unclear, or contradictory.

In EHR systems that lack discrete Sig-builder functionalities and only provide free-text fields for manual entry of patient directions, development of system and user-interface enhancements, including implementation and appropriate use of Sig-builder tools to compose standardized Sig strings, could improve the quality of prescriptions along with efficiency and end-user experiences. Moreover, EHR system vendors that already provide discrete Sig-builder tools should develop further enhancements and refinements to better accommodate the generation of more complete Sigs for the specific drug formulations prone to QREs. Since Sigs for topical products were associated with the highest prevalence of QREs, the majority of which related to a missing dose or site of administration, additional focus should be placed on ensuring Sig-builder tools provided by e-prescribing applications require the selection of explicit dose amounts in the Sig (e.g., pea-size amount, thin layer, sparingly, 1-inch strip, etc.) and specified sites of administration (e.g., on face, on right leg, on wound, on affected area, etc.) before prescribers complete and transmit the order.

Individual preferences in prescriber behaviors and habits are another possible contributor to the significant variations and quality-improvement opportunities that persist in freetext Sig strings. Therefore, e-prescribing software system and interface refinements should also be accompanied by increased end-user training and usability testing to ensure full familiarity with, and optimal use of, all application tools and functionalities. Continuous user feedback should be solicited, and thorough consideration should be given for specific challenges reported by prescribers.^19^

Although our results suggest that the most commonly used Sigs can be represented by a relatively small number of standard Sig text strings, the sheer number and diversity of needs that prescribers have for issuing clear directions to patients suggest a continuing need for access to free-text Sig options in e-prescribing. Thus, e-prescribing applications will still need to provide the capability for manual entry of highly complex directions on the rare occasion that the Sig cannot be accommodated by even the most robust Sig-builder tools.

However, it is recommended that e-prescribing technology vendors minimize the need for manual typing of the entire Sig by prescribers. One solution is further development of enhanced clinical decision support tools that provide prebuilt complete Sig strings that are accurate and relevant. By creating these default strings for the most frequently used patient instructions associated with the most commonly prescribed medications, end-users would simply need to choose a preconstructed complete Sig, thereby minimizing laborious manual entry and decreasing the potential for Sig QREs from entry errors.

Finally, another contributor to e-prescription quality issues is the absence of consistent, industry-wide implementation of standardized Sig information and its subsequent transmission in machine-readable formats, hampering computerized clinical decision support checks.^12^ One solution here is the adoption and implementation of the Structured and Codified Sig format available in the current NCPDP SCRIPT Standard version 10.6. Through codification, EHRs and pharmacy systems could map individual Sig components that have the same semantic meaning or are synonymous, regardless of their textual string variations, to Systematized Nomenclature of Medicine—Clinical Terms (SNOMED CT) codes. SNOMED CT is a robust, standardized vocabulary of clinical terminology established by the National Library of Medicine and organized in discrete fields in the e-prescription.^9^ Recipient systems of SNOMED CT codes in discrete fields could then process and translate the codes back to text and construct complete and standardized patient instructions for expedient e-prescription processing. This functionality would subsequently facilitate improved system interoperability, clinical-decision support safety checks on discrete codified data that are not possible on free-text strings, and more seamless workflows that minimize manual human interventions such as retyping.

Since codification of just 25 of the most common Sig concepts in accordance with the uniform format established by the SCRIPT standard would help standardize more than half of Sigs typically transmitted in the ambulatory care setting, pharmacy and EHR applications electing to implement this feature would gain the most sizable impact on the majority of their e-prescriptions with the lowest amount of system development complexities and coding efforts.

Furthermore, as the e-prescribing standard continues to develop and advance, the Structured and Codified Sig segment would also evolve in tandem to further accommodate many of the more complex and less common Sig concepts for future developments. A significant barrier to this approach is that Structured and Codified Sig is currently an optional segment in e-prescriptions and has seen minimal use across the industry. Additional industry-wide education and e-prescribing application system developments from both the prescriber EHR vendors and the pharmacies will therefore be necessary to drive adoption and meaningful bidirectional interoperability.

### Limitations

Several limitations of this study should be noted. First, the data analyzed were limited to a random sample of only new e-prescriptions in the ambulatory setting transmitted on a single day. Thus, the content within the study sample may not be entirely representative of all possible e-prescriptions, as the types of prescribed products and drug formulations may be significantly different in other settings such as acute-care institutions or long-term-care facilities. Consequently, the Sigs for these products may have different challenges and QREs.

Second, while the Sigs with the highest QRE rates were observed for topical medications, quantifying additional trends in the patient directions associated with QREs for every possible drug formulation was outside the scope of this analysis. Thus, a more comprehensive study should be conducted to analyze prescription orders from other types of settings and for a wider variety of dosage formulations to obtain a more comprehensive picture of the opportunities and quality challenges in their different Sigs.

Third, this study did not investigate Sig quality trends that may be present in nonmedication products such as durable medical equipment or testing supplies. These products, while often e-prescribed, may not have instructions that follow a comparable syntax as medications. For example, devices, diabetic test strips, and crutches or braces would logically not have the core dose component or routes of administration as do medications.

Fourth, no QRE analyses were performed for quality trends in Sig concepts present in fewer than 20 e-prescriptions. It is possible that several quality issues with greater complexity may be present in the more unique Sig concepts, some of which may involve multiple combinations of QREs. However, since these infrequently used Sig concepts appeared in so few e-prescriptions, their distribution was considered insufficient to draw meaningful conclusions.

Finally, while the pharmacy technicians who reviewed e-prescriptions for QREs were experienced in the ambulatory care setting and had received additional specific training and assessment to identify potential problems, some Sigs may contain subtle nuances that require the clinical background and training of a pharmacist to discern. The percentage of e-prescriptions judged to contain 1 or more QREs should therefore be considered a conservative estimate of the actual prevalence of QREs in e-prescriptions.

## Conclusions

Free-text patient directions in e-prescriptions can vary dramatically, with up to 832 permutations of text strings to convey a single Sig concept. Despite this variance, more than half of all patient directions sent in the ambulatory setting can be categorized into just 25 Sig concepts, suggesting an immediate, practical opportunity for improving communication between prescribers and pharmacies. In addition to textual string variance, more than 1 in 10 e-prescriptions contained a quality issue in the Sig that would likely require pharmacy personnel to contact prescribers for clarification or other manual interventions that create workflow disruptions. These quality issues may also pose potential patient safety risks if the Sig is misinterpreted by pharmacy staff and the prescriber’s intent is not fully or accurately conveyed to the patient.

Recommendations to address the quality issues, variations, and inconsistencies present in Sig strings include enhancing e-prescribing application user interfaces and Sig creation tools, improving end-user training and usability testing for optimal use of system functionalities, and adopting and implementing the currently available Structured and Codified Sig format by both prescriber and pharmacy systems to facilitate improved standardization and interoperability.

Given the legislative efforts, regulatory mandates, and extensive financial incentives that have been dedicated toward promoting the adoption, implementation, and meaningful usage of e-prescribing, improvements in how Sigs are written, communicated, and processed represents a logical next step toward realizing the full potential benefits of e-prescribing for prescribers, pharmacies, and patients.

## APPENDIX Sig Content Concept Classification Scheme

Sig free-text strings were classified with a concept code intended to capture the directions for the patient and based on the intended clinical meaning of the instructions. For example, the Sig concept of “take one tablet by mouth once daily” may be communicated by free-text in the following variations: “Take 1 tablet daily orally,” “1 (one) tablet(s) by mouth once a day,” “Take 1 tablet every day,” or “1 tab(s) PO daily.” Since the meaning behind these strings is all the same, a singular concept code of “T1TQD” was assigned to categorize the Sigs.

The basic format and sequence for the concept codes follows the basic format and sequence found in most of the Sigs and included the following core elements: Action, Dose, Dose Unit, and Frequency, followed by auxiliary information. In cases where the original string had an element missing, such as the dose formulation or route of administration, the reviewer examined the drug description sent in the message associated with the Sig to determine the appropriate associated element. For example, if a Sig string stated only “Take 1 daily” and the associated drug description was “Atorvastatin 20 mg oral tablet,” the reviewer logically inferred the dose form of “tablet” as well as the route of administration of “oral” when assigning the concept code.

Some patient directions included additional variances in elements such as the duration of therapy or indications. Since duration of therapy values could vary from hours to months, concept codes included an “X” as a placeholder to represent a numerical duration value, thereby minimizing the number of concept code variants within a similar theme. For example, “take 1 tablet twice a day orally for 10 day(s),” “1 tab(s) BID oral 90 days,” and “Take 1 tablet(s) by mouth given 2 times a day for 14 days” would all be assigned the classification code “T1TBID X” to convey the concept of “take one tablet by mouth twice daily for a specified duration.”

Similarly, the term “FOR” was used as a placeholder in Sig concept codes to represent some indication, condition, or therapeutic goal for which the prescribed drug was intended. For example, “Take 1 capsule daily for tremors,” “1 cap oral daily for reflux,” and “Take 1 capsule po daily for acne” were all assigned the concept code of “T1CQD FOR” to convey that the medication was prescribed for some specific indication. Likewise, if the frequency indicated “as needed” (PRN) and the Sig included a specific reason, the concept code included the term “PRNY” to indicate a particular medical reason, condition, or therapeutic goal had been specified.
